# Calreticulin down-regulation inhibits the cell growth, invasion and cell cycle progression of human hepatocellular carcinoma cells

**DOI:** 10.1186/s13000-015-0382-1

**Published:** 2015-08-27

**Authors:** Ruo Feng, Jianwen Ye, Chuang Zhou, Lei Qi, Zhe Fu, Bing Yan, Zhiwei Liang, Renfeng Li, Wenlong Zhai

**Affiliations:** Department of Histology and Embryology, Medical College of Zhengzhou University, Zhengzhou, People’s Republic of China; Department of Hepatobiliary and Pancreatic Surgery, the First Affiliated Hospital of Zhengzhou University, Zhengzhou, People’s Republic of China; Key Laboratory of Hepatobiliary and Pancreatic Surgery & Digestive Organ Transplantation of Henan Province, Zhengzhou, People’s Republic of China; Department of Endocrine, the First Affiliated Hospital of Zhengzhou University, Zhengzhou, People’s Republic of China

## Abstract

**Background:**

Hepatocellular carcinoma (HCC) is one of the most frequent cancers in the world. Calreticulin(CRT) is aberrantly overexpressed in many human cancer cells. The function of CRT in HCC cells remains unclear. We attempted to investigate the effects and the underlying mechanisms of CRT down-regulation on HCC cell growth, apoptosis, cell cycle progression and invasion.

**Methods:**

To investigate the function of CRT in HCC cells, small interfering RNA (siRNA) was used to knock down the expression of CRT in SMMC7721 and HepG2 HCC cells. CRT expression was examined by Western blot and immunofluorescence. Cell proliferation was detected by CCK-8 assay. Cell cycle and apoptosis were measured by the flow cytometry. The invasion capability was assessed by transwell assay. The phosphorylation level of Akt was evaluated by Western blot.

**Results:**

Compared with human hepatic cells L02, CRT was apparently up-regulated in SMMC7721, HepG2 and Huh7 HCC cells. Down-regulation of CRT expression effectively inhibited HCC cell growth and invasion. CRT knockdown induced cell cycle arrest and the apoptosis in SMMC7721 and HepG2 cells. Furthermore, down-regulation of CRT expression significantly decreased the Akt phosphorylation.

**Conclusions:**

CRT was aberrantly over-expressed in HCC cell lines. CRT over-expression contributes greatly to HCC malignant behavior, likely via PI3K/Akt pathway. CRT could serve as a potential biomarker and therapeutic target for hepatocellular carcinoma.

## Background

Hepatocellular carcinoma (HCC) is the most common primary liver malignancy with a high rate of metastasis and recurrence. It is the sixth most common malignancy worldwide and the third cause of cancer-related mortality [1, 2]. Although new progresses have been made in the surgical techniques, transcatheter arterial chemotherapy (TACE), radiotherapy, chemotherapy and liver transplantation, the prognosis of HCC remains poor. To make an early diagnosis and to improve the survival of HCC patients, new efficient biomarkers and molecular therapeutic targets need to be sought.

Calreticulin (CRT) is a multi-functional molecular chaperone mostly residing in endoplasmic reticulum and plays an important role in regulating biological processes, such as Ca^2+^ homeostasis, transcriptional regulation, immune response and cellular functions including cell proliferation, migration, adhesion and apoptosis, etc. [3, 4]. CRT is located on chromosome 19p13 and its promoter region contains kinds of regulatory sites such as AP-1,AP-2 and H4TF-1 [3, 5]. A number of transcription factors have been found to modulate CRT gene, which plays a critical role in tumor development and pathological progression [5]. CRT protein consists of the N-terminal, C-terminal and three different domains in between. The N-terminal is a cleavable amino acid signal sequence which is responsible for its biological function such as chaperoning and Ca^2+^-buffering, while the C-terminal contains endoplasmic reticulum retrieval signals [3, 5]. Recently, CRT was shown to be highly expressed in multiple kinds of human cancers, including pancreatic cancer, colon cancer, oral squamous cell carcinoma and gastric carcinoma [6–9]. It has been shown that CRT expression is closely related to the tumor progression, metastasis and the poor prognosis in both esophageal cancer [10] and breast cancer [11]. Lu et al. have shown that knockdown of CRT inhibited cell proliferation and migration via FAK pathway in the bladder cancer. In vivo data showed that knockdown of CRT led to fewer metastatic sites in the lung and liver [12]. Over-expression of CRT facilitated cell proliferation and migration and modulated several molecules related to cancer metastasis and angiogenesis in gastric cancer [13]. Other evidences indicated that endoplasmic reticulum stress mediated immunity of tumor cell vaccine via the CRT translocation to the cell membrane [14]. It was also demonstrated that CRT is required for TGF-βstimulated extracellular matrix (ECM) production which provided a link between enhanced endoplasmic reticulum stress and TGF-β stimulated ECM production [15]. The role of CRT in the HCC remained unclear.

To explore the effects of CRT on the tumor biological phenotypes in HCC cells, SMMC7721 and HepG2 HCC cells were transfected with the small interfering RNA targeting CRT. The effects of CRT down-regulation on cell proliferation, invasion, cell cycle progression, apoptosis and its possible underlying molecular mechanisms were studied.

## Methods

### Materials

The human hepatocellular carcinoma cell lines (SMMC7721、HepG2 and Huh7 cells) and human normal hepatic cells (L02) were purchased from shanghai cell bank (China Academy of Science) and cultured in DMEM medium (Hyclone) supplemented with 10 % fetal bovine serum (Gibco USA), 100 units/ml penicillin and 100 mg/L streptomycin (Sigma) under a humidified atmosphere of 5 % CO_2_ at 37 °C.

### Transfection

siRNA for CRT was synthesized by GenePharma Biotechnology (Shanghai, China). SMMC7721 and HepG2 cells were cultured in a complete medium without antibiotics. For RNAi experiment group, 5 μl of lipofectamine-2000 was diluted in 100 μl of DMEM for 5 min at room temperature. Then 5 μl of siRNA (20 μM) was mixed with DMEM containing Lipofectamine-2000 and incubated for 20 min at room temperature for the complex formation. Finally, the complex was added to the wells containing 2 ml medium with 100 nM final siRNA concentration. CRT protein expression was determined by both Western blot and immunofluorescence 36 h after transfection. The siCRT sequences were 5’-GCACCAUCUUUGACAACUUTT-3’ (sense) and 5’-AAGUUGUCAAAGAUGGUGCTT -3’ (antisense). The sequences of the negative control were 5’-UUCUCCGAACGUGUCACGUTT-3’ (sense) and 5’-ACGUGACACGUUCGGAGAATT-3’ (antisense).

### Western blot

Whole cell lysates were extracted with RIPA buffer (10 mmol/l Tris–HCl, pH8.0, 10 mmol/l EDTA, 0.15 mol/l NaCl, 1 % NP-40, 0.5 % sodium dodecyl sulphate, 1 μg/ml Aprotinin, 1 mmol/l phenyl methyl sulphonyl fluoride) on ice for 30 min. Protein supernatant was collected by centrifugation at 15,000 g for 15 min. Protein concentration was determined by the Bio-Rad protein assay (Bio-Rad Laboratories, Hercules, CA, USA). Each extract containing approximately 30 μg protein was subjected to 10 % polyacrylamide gel electrophoresis and transferred onto polyvinylidene difluoride (PVDF) membranes (MilliPore, Bedford, MA, USA). The membranes were blocked in TBST (5 mmol/l Tris–HCl, pH 7.4, 136 mmol/l NaCl, 0.1 % Tween 20) containing 5 % nonfat dry milk for 2 h at room temperature, and hybridized with the primary antibody (CRT (Abcam ab22683 ) at 1:3000 dilution, phospho-Akt(Ser473) (D9E) (Cell Signal Technology ) at 1:1000 dilution, Akt(pan) (C67E7) (Cell Signal Technology ) at 1:1000 dilution) overnight at 4 °C, followed by three washes for 5 min with TBST. Then the membranes were incubated with HRP-conjugated goat anti-rabbit IgG (dilution 1:5000) for 1 h at room temperature and washed three times with TBST. Blots were detected using the enhanced chemiluminescence western blotting detection kit (Amersham Biosciences, Piscataway, NJ, USA) according to the manufacturer’s instruction. β-actin served as the loading control.

### Immunofluorescence assay

SMMC7721 and HepG2 cells were seeded into the 6-well plates and transfected with siRNA for 36 h. After treatment, SMMC7721 and HepG2 cells were fixed with 70 % methanol for 20 min, and blocked with 5 % BSA for 1 h at room temperature and then incubated with the mouse anti-calreticulin antibody (1:100 dilution) overnight at 4 °C,followed by three washes for 5 min with TBST. Then the cells were incubated with the Alexa Flour 555-conjugated anti-mouse secondary antibody (1:100 dilution) for 1 h and the CRT immunofluorescence was observed using fluorescence microscope (Leica, Germany). DAPI was used to show the nuclei.

### CCK-8 assay

Cell viability assay was detected by the CCK-8 assay. SMMC7721 and HepG2 cells were seeded into the 96-well plates at a density of 1000 cells/well and incubated for 0、24、36、48 h, respectively. Then the cells were incubated with 10 μL CCK-8 solution for 2 h. The OD values were measured using the absorbance micro-plate reader (Thermo) at a wavelength of 450 nm.

### Transwell invasion assay

SMMC7721 and HepG2 cells were resuspended in serum-free DMEM and seeded in the matrigel-coated insert on the top portion of the chamber (BD Bioscience). The lower compartment of the chamber contained 10 % FBS as a chemo-attractant. After incubated for 24 h, the cells on the membrane were scrubbed, washed with PBS and fixed in 100 % methanol for 20 min and stained with 0.1 % crystal violet for 10 min. The cells invaded through the membrane were visualized with an inverted microscope (Olympus).

### Flow cytometry analyses

For the cell cycle analysis, SMMC7721 and HepG2 cells were collected and fixed in 70 % cold ethanol overnight at 4 °C, followed by the incubation with propidium iodide (PI) solution for 30 min in dark at 37 °C,then were analyzed by the flow cytometry. For cell apoptosis analysis, cells were collected and added Annexin V/FITC, propidium iodide followed by incubation in dark at room temperature for 10 min. Apoptosis was the detected by flow cytometry.

### Statistical analysis

SPSS version 17.0 was used for statistical analysis. All data were expressed as mean ± SD of at least three independent experiments. One-way analysis of variance was used to analyze the data. *P* < 0.05 was considered to be statistically significant.

## Results

### The expression level of CRT in hepatic cells and HCC cells and the effects of siRNA on the CRT expression level

**Fig. 1:**
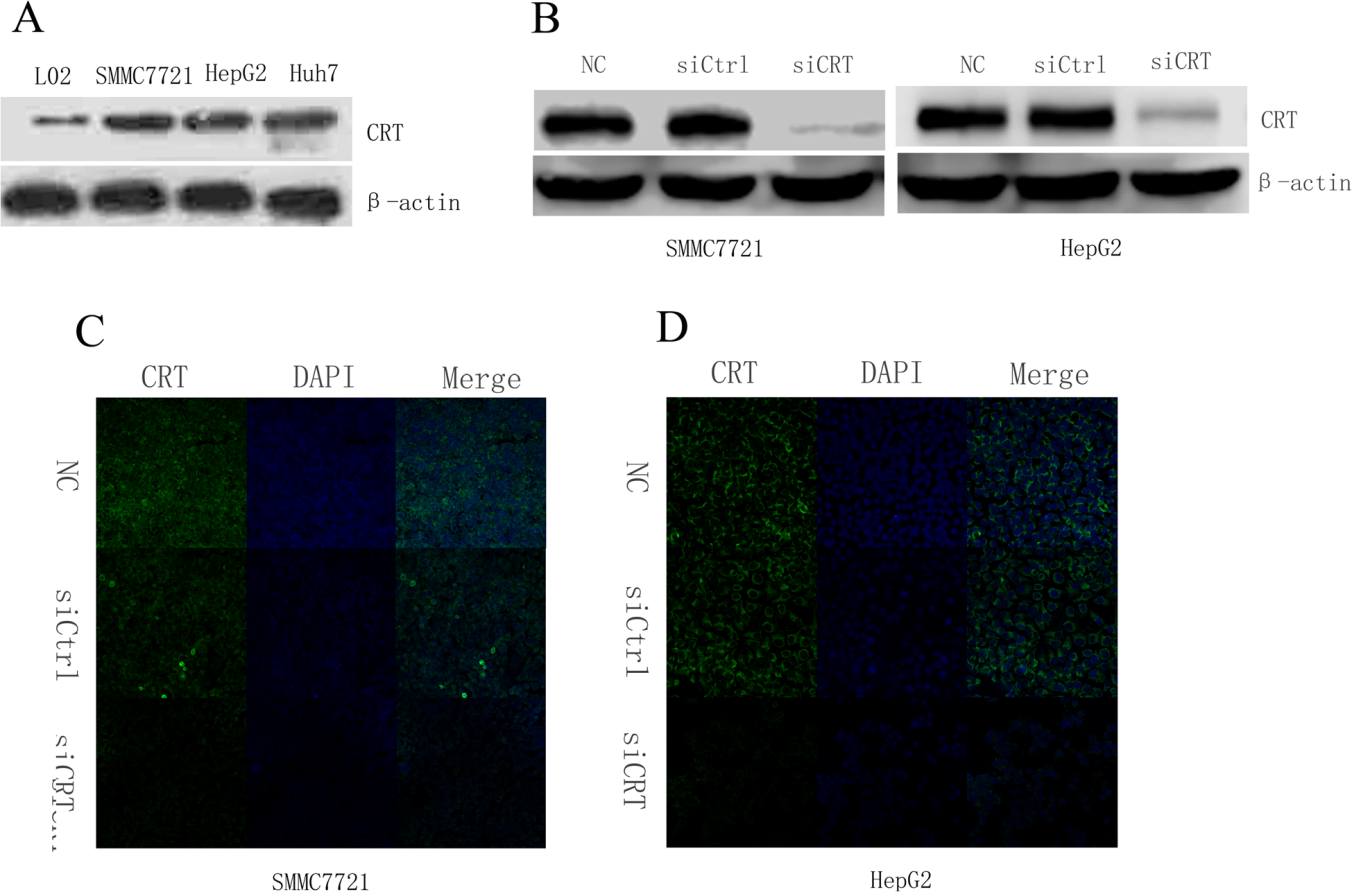
Differences in the CRT expression levels between hepatic cells and HCC cells and the effects of siRNA on CRT expression. **a**. Whole cell lysate from indicated HCC cells and L02 hepatic cells were subjected to Western blot analysis. β-actin served as a loading control. **b**. The expression of CRT in SMMC7721 and HepG2 cells was detected by Western blot analysis after transfected with CRT siRNA and negative control siRNA for 36 h. **c**. SMMC7721 cells were transfected with CRT siRNA and negative control siRNA for 36 h. The expression of CRT was detected by immunofluorescence. SMMC7721 without any transfection served as a negative control. **d**. HepG2 cells were transfected with CRT siRNA and negative control siRNA for 36 h. The expression of CRT was detected by immunofluorescence. HepG2 without any transfection served as a negative control. Figures shown are representatives of at least 3 independent experiments

CRT is closely related with tumorigenesis and tumor progression [6]. The expression of CRT in multiple HCC cell lines and the L02 hepatic cells were examined. As shown in Fig. 1a, the protein levels of CRT in SMMC7721, HepG2 and Huh7 HCC cells were significantly higher than that in L02 hepatic cells. This result indicates that CRT is aberrantly over-expressed in HCC cells. To test the role of CRT in HCC tumorigenesis and progression, small interfering RNA was transfected into SMMC7721 and HepG2 HCC cells. Western blot data showed obviously decreased CRT expression levels in CRT siRNA (siCRT) group in both SMMC7721 and HepG2 HCC cells comparing to the negative control (NC) and the siRNA control (siCtrl). CRT expression level in NC group in both SMMC7721 and HepG2 was similar to that in siCtrl group (Fig. 1b). Similar results were obtained using immunofluorescence assay (Fig. 1c and d). These results demonstrate that CRT siRNA can effectively down-regulate the expression of CRT protein in both SMMC7721 and HepG2 cells.

### Down-regulation of CRT inhibits the cell proliferation in HCC cells

**Fig. 2:**
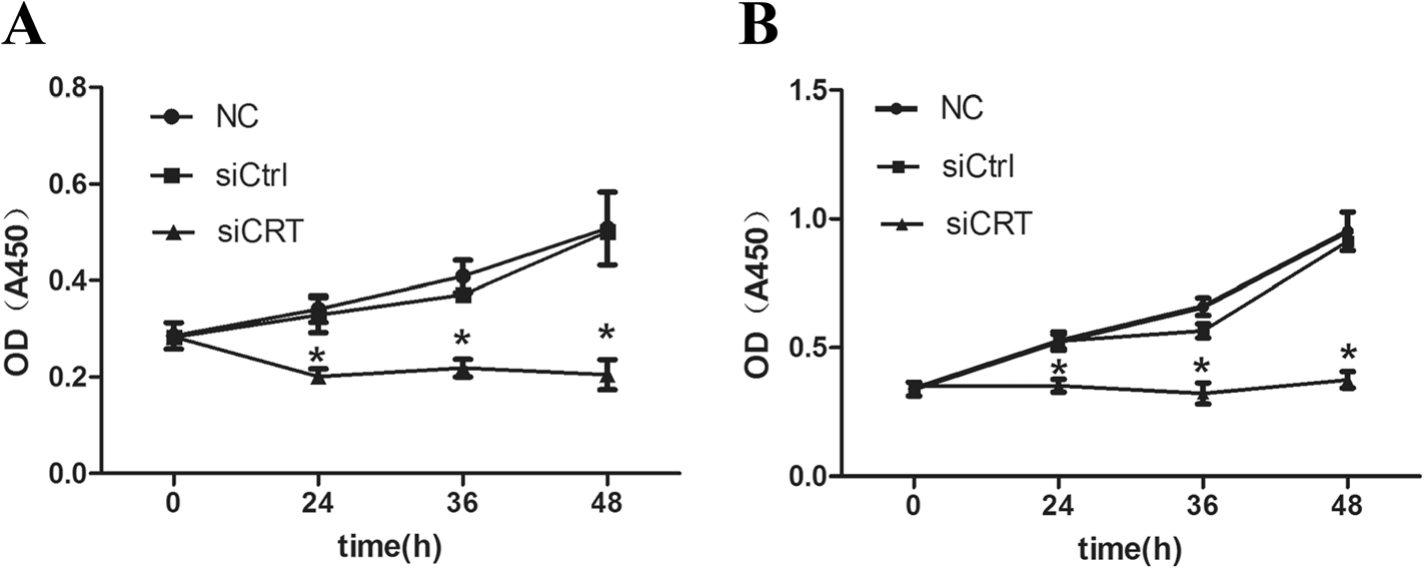
Down-regulation of CRT inhibits the cell proliferation in HCC cells. **a**. SMMC7721 cells were transfected with CRT siRNA and negative control siRNA (siCtrl) for indicated periods. Proliferation was measured by CCK-8 assay. SMMC7721 without any transfection served as a negative control (NC). **b**. HepG2 cells were transfected with CRT siRNA and negative control siRNA for indicated periods. Proliferation was measured by CCK-8 assay. HepG2 without any transfection served as a negative control (NC). Data were shown as the mean ± SD of at least three independent experiments. Statistical differences were compared with the siCtrl and NC (*P <0.05)

CCK-8 assay was used to measure the cell growth and proliferation. As seen in Fig. 2a, significantly growth inhibition was observed in SMMC7721 cells transfected with siCRT (*P < 0.05*). The relative growth inhibition rate was (41.0 ± 2.2)%, (46.5 ± 1.6)% and (59.7 ± 2.2)% at 24 h, 36 h and 48 h, respectively. The proliferation ability in siCtrl group was similar to that in NC group. Similar results were obtained for HepG2 cells, with the relative growth inhibition rate as (36.8 ± 2.7)%, (47.3 ± 1.8)% and (61.5 ± 3.2)% at 24 h, 36 h and 48 h, respectively.

### Down-regulation of CRT induces the cell apoptosis in HCC cells

**Fig. 3:**
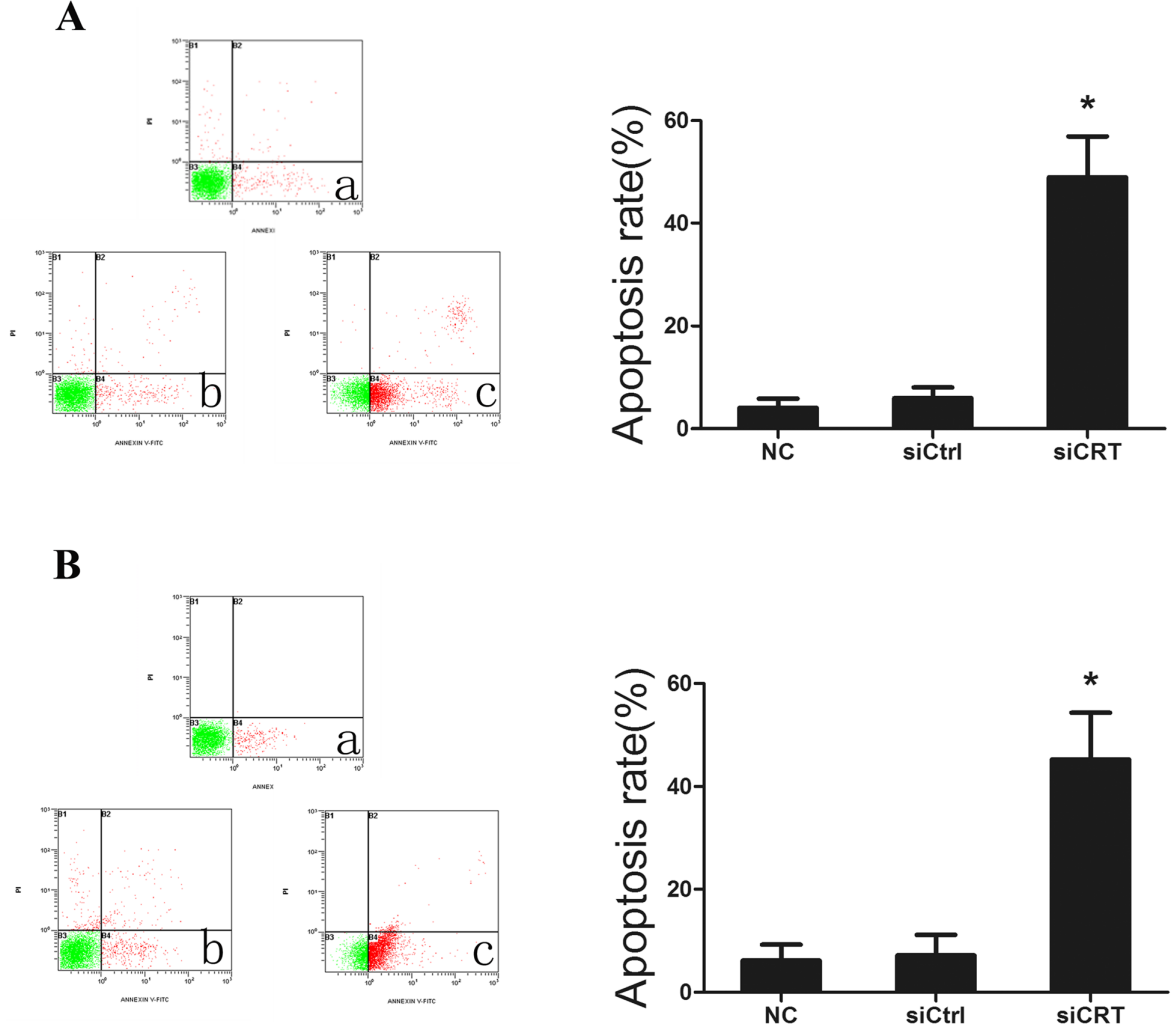
Down-regulation of CRT induces the cell apoptosis in HCC cells. **a**. SMMC7721 cells were transfected with CRT siRNA and negative control siRNA for 36 h. The apoptosis was examined by Annexin V/FITC and propidium iodide staining method using flow cytometry. SMMC7721 without any transfection served as a negative control. SMMC7721 apoptotic rates were quantified. Each bar of the histogram represents the quantified data shown as the mean ± SD of at least three independent experiments. **b**. HepG2 cells were transfected with CRT siRNA and negative control siRNA for 36 h. The apoptosis was examined by Annexin V/FITC and propidium iodide staining method using flow cytometry. HepG2 without any transfection served as a negative control. HepG2 apoptotic rates were quantified. Each bar of the histogram represents the quantified data shown as the mean ± SD of at least three independent experiments. Figures shown are representatives of at least 3 independent experiments. Statistical differences were compared with the siCtrl and NC (*P <0.05)

Flow cytometry was employed to measure the apoptosis in SMMC7721 and HepG2 cells. As shown in Fig. 3a, SMMC7721 cell apoptotic rate of NC group and siCtrl group was (4.0 ± 2.1)% and (5.9 ± 1.5)%, respectively. Down-regulation of CRT effectively increased the apoptotic rate to (45.2 ± 9.1)% (*P < 0.*05*)*. As shown in Fig. 3b, similar results were obtained for HepG2 cells. The apoptotic rate of NC group and siCtrl group was (6.2 ± 1.8)% and (7.2 ± 1.4)%, respectively; while the apoptosis level for the knockdown group is increased to (45.2 ± 9.1)% (*P < 0.*05*)*. These data demonstrated that down-regulation of CRT can effectively induce apoptosis in HCC cells.

### Down-regulation of CRT induces cell cycle progression arrest in HCC cells

**Fig. 4:**
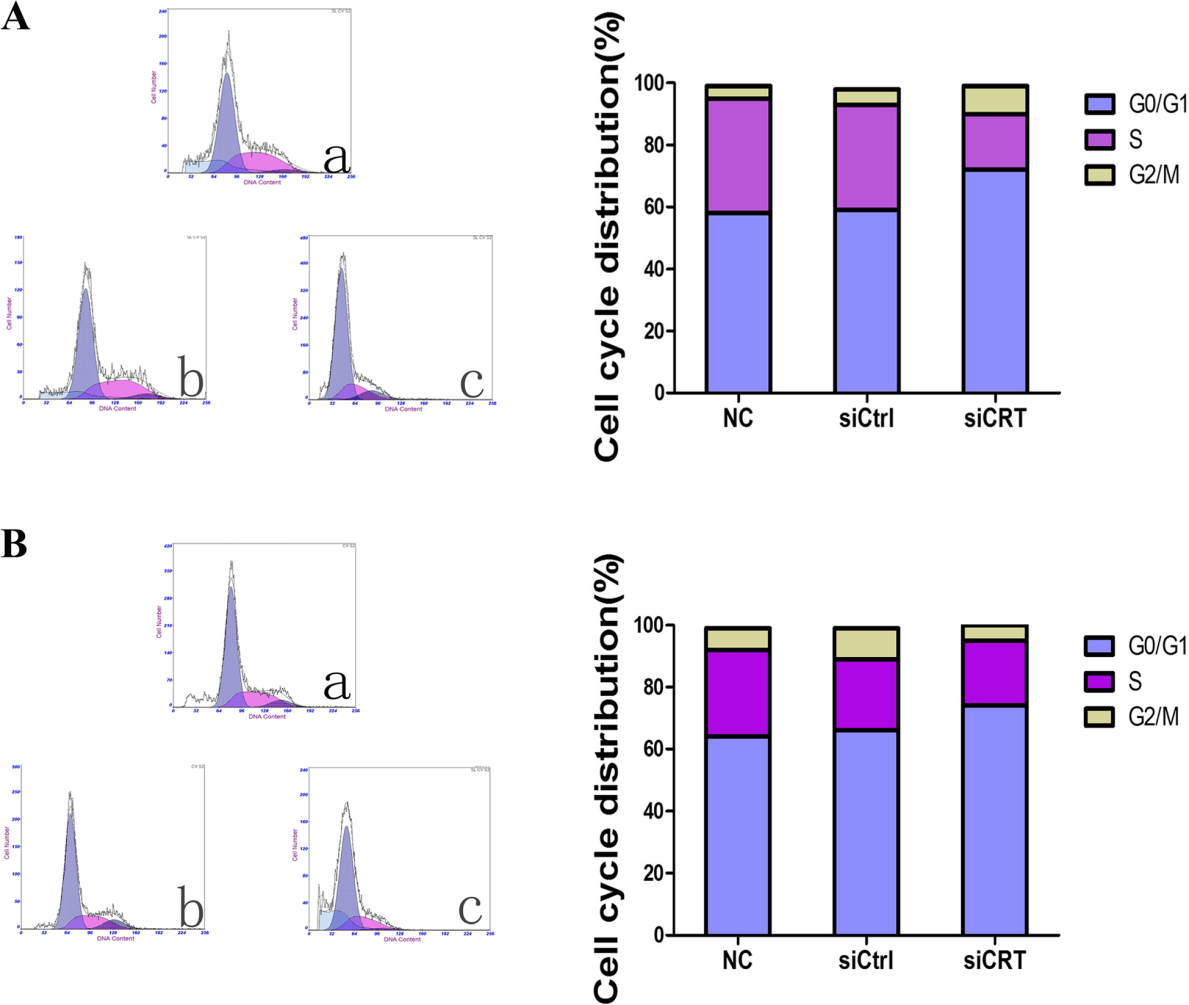
Down-regulation of CRT induces cell cycle progression arrest in HCC cells. **a**. SMMC7721 cells were transfected with CRT siRNA and negative control siRNA for 36 h. Cell cycle progression was analyzed by propidium iodide staining method using flow cytometry. SMMC7721 without any transfection served as a negative control. The percentages of SMMC7721 cells in each phase were quantified. **b**. HepG2 cells were transfected with CRT siRNA and negative control siRNA for 36 h. Cell cycle progression was analyzed by propidium iodide staining method using flow cytometry. HepG2 without any transfection served as a negative control. The percentages of HepG2 cells in each phase were quantified. Figures shown are representatives of at least 3 independent experiments. Statistical differences were compared with the siCtrl and NC (*P <0.05)

The effects of CRT knockdown on the HCC cell cycle progression were evaluated by the flow cytometry analysis. As shown in Fig. 4a, the percentage of cells in G0/G1 phase was significantly higher in siCRT group (72 %) in SMMC7721 cells comparing to NC group and siCtrl group (59 %) (*P < 0.*05*)*. Similar results were obtained for HepG2 cells as shown in Fig. 4b. These data indicate that down-regulating CRT can induce cell cycle arrest in HCC cells.

### Down-regulation of CRT inhibits the invasive ability of HCC cells

**Fig. 5:**
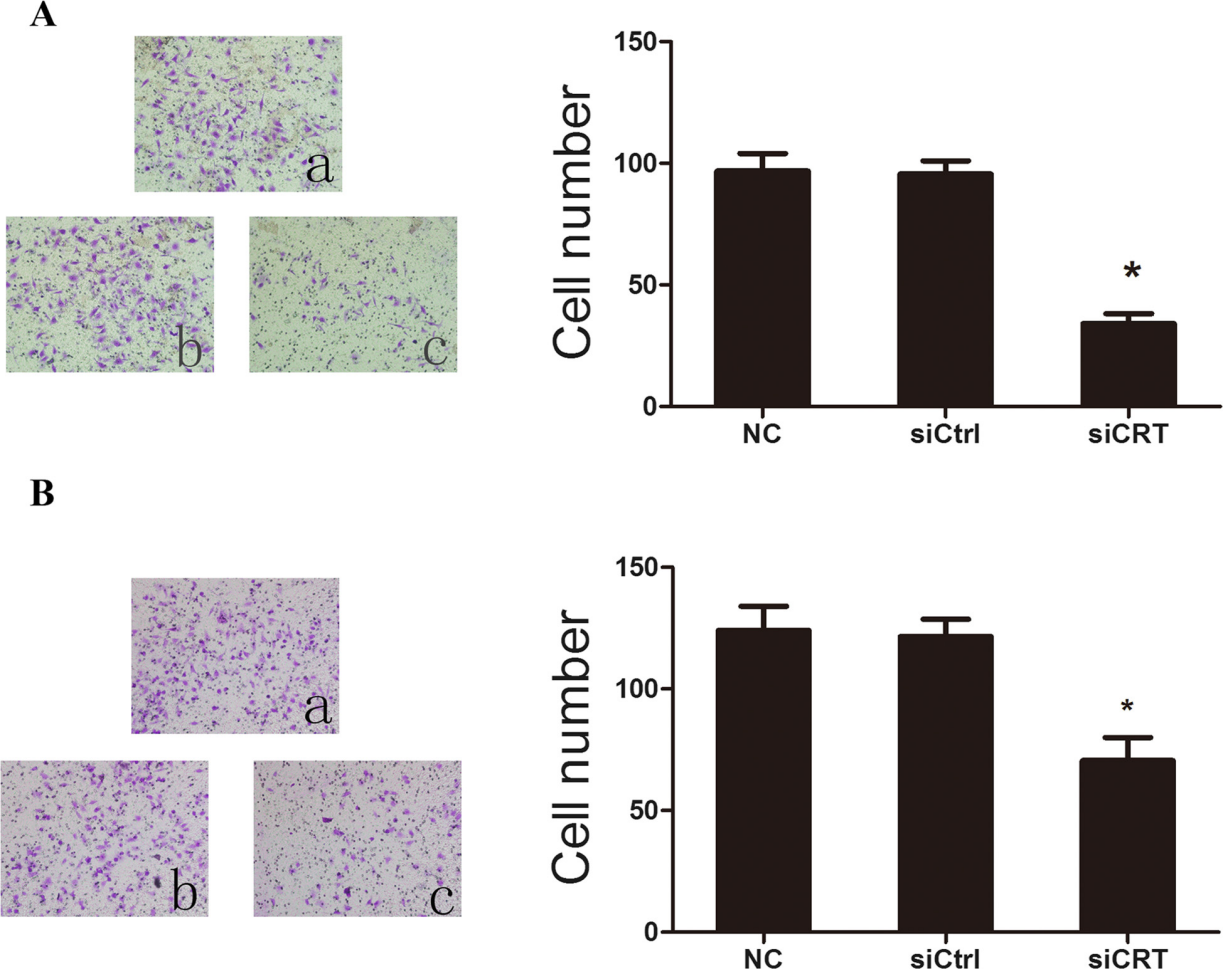
Down-regulation of CRT inhibits the invasive abilities of HCC cells. **a**. SMMC7721 cells were transfected with CRT siRNA and negative control siRNA for 36 h. The invasive abilities were determined by transwell assay. SMMC7721 without any transfection served as a negative control. SMMC7721 invasive abilities were quantified. Each bar of the histogram represents the number of the invaded cells per high power field. Data are shown as the mean ± SD of at least three independent experiments. **b**. HepG2 cells were transfected with CRT siRNA and negative control siRNA for 36 h. The invasive abilities were determined by transwell assay. HepG2 without any transfection served as a negative control. HepG2 invasive abilities were quantified. Each bar of the histogram represents the number of the invaded cells per high power field. Data are shown as the mean ± SD of at least three independent experiments. Figures shown are representatives of at least 3 independent experiments. Statistical differences were compared with the siCtrl and NC (*P <0.05)

The effects of CRT knockdown on the HCC cell invasion were tested by the transwell invasion assay. Figure 5a shows that (34.0 ± 4.2) cells per high power field invaded through matrigel in siCRT knockdown group of SMMC7721 cells, which is significantly less than that (96.8 ± 7.3) in NC group and (95.6 ± 5.4) siCtrl group (*P < 0.*05*)*. Similar results were observed forHepG2 cells (Fig. 5b): (70.4 ± 9.5) cells per high power field have invaded through matrigel in siCRT knockdown group of HepG2 cells, which was significantly less than that (124.0 ± 9.9) in NC group and (121.6 ± 7.0) siCtrl group (*P < 0.*05*)*. These results demonstrated that down-regulation of CRT could inhibit cell invasion in HCC cells.

### Down-regulation of CRT inhibits the activation of PI3K/Akt pathway

**Fig. 6:**
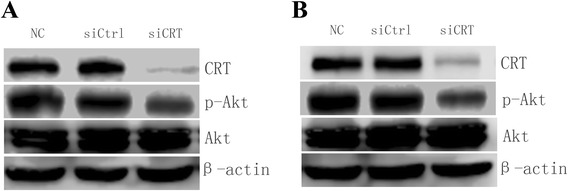
Down-regulation of CRT inhibits the activation of PI3K/Akt pathway. **a**. Whole cell lysate from SMMC7721 with or without indicated transfection for 36 h were subjected to Western blot analysis. β-actin served as a loading control. **b**. Whole cell lysate from HepG2 with or without indicated transfection for 36 h were subjected to Western blot analysis. β-actin served as a loading control. Figures shown are representatives of at least 3 independent experiments

PI3K/Akt pathway regulates tumor cell proliferation, apoptosis, cell cycle progression and invasion in many types of carcinomas [16–18]. To study the underlying mechanisms of CRT-associated tumorigenesis and progression, the activation status of Akt signaling in SMMC7721 and HepG2 were monitored. Figure 6 shows that the phosphorylation of Akt was markedly suppressed by knocking down CRT in HCC cells. SMMC7721 and HepG2 transfected with CRT siRNA showed lower phosphorylation levels of Akt than those in the controls, while no significant difference in the Akt expressionwas observed in all the groups. These data suggest that CRT might regulate the tumor biological phenotypes by activating PI3K/Akt pathway via Akt phosphorylation.

## Discussion

Hepatocellular carcinoma is an aggressive malignancy that exerts huge burdens on patients and the health care systems. Rapid tumor progression and difficulty in detecting early disease are major obstacles in offering potentially curative treatments. Currently, there are no reliable tumor markers for diagnosing HCC at early stage. No effective ways are available for predicting disease progression. In this study, we showed that human HCC cell lines aberrantly over-express CRT as compared to that in non-malignant human hepatic cells. Down-regulation of CRT can inhibit HCC cell proliferation and invasion, induce apoptosis and hault cell cycle progression. Mechanistically, CRT knockdown suppresses phosphrylation of Akt, thus potentially inactivate the PI3K/Akt pathway. CRT over-expression may serve as a potential biomarker for HCC detection; CRT may also serve as a potential therapeutic target.

Previous reports have shown that CRT is up-regulated in other cancers. Close correlation between CRT expression and metastasis was observed [6, 19, 20], suggesting CRT is probably a pivotal molecular signal for cancer growth and progression. Yoon et al. demonstrated that CRT is highly expressed in HCC patients’ samples when compared to noncarcinomatous hepatic tissues. In the noncarcinomatous tissues, CRT is distributed extensively only in the cytoplasm of hepatocytes and Kupffer cells. CRT show both cytoplasm and nuclear localization in the HCC cells [21]. These data indicate that not only the level of expression but the localization of CRT might play important roles in HCC tumor progression.

Rapidly proliferating cancer cells demands increased protein synthesis. Therefore, enhanced endoplasmic reticulum (ER) activity is required to facilitate the folding, assembly and transportation of membrane and secretory proteins. These functions are carried out by ER chaperones. It is now becoming increasely clear that chaperons play important roles in cell function in addition to simply facilitating protein folding [22]. As a multi-functional endoplasmic reticulum chaperon, it was found that over-expression of CRT contributes to the tumor development and progression, promotes migration and invasion, and is closely correlated with the poor prognosis [6]. Kabbage et al. found that CRT is over-expressed in infiltrating ductal breast carcinoma specimens. However, statistical analyses revealed no significant correlations between calreticulin expression and clinicopathological parameters of the disease including tumor stage, SBR grade, and lymph node metastasis occurrence [20]. Therefore, it is necessary to define the role of CRT over-expression in HCC carcinogenesis and progression. More studies are needed to clarify these issues in the clinical settings.

PI3K/Akt signaling pathway is highly activated in HCC and also contributes to HCC proliferation, invasion, and anti-apoptosis,etc. [17, 23]. It has been reported that CRT can serve as an upstream signal for the activation of PI3K/Akt [24, 28]. In the CRT over-expressing cells, phospho-Akt is up-regulated [24]. Akt activation accelerates tumor cell migration and invasion [25–28]. In addition, it was shown that CRT can foster tumor cell migration and enhance resistance to anoikis via activation of the PI3K/Akt signaling pathway [28]. Although most of the studies showed that CRT up-regulation leads to Akt activation, Liu et al. reported that CRT over-expression inhibited Akt phosphorylation in non-small cell lung cancer cells [29]. Therefore, the CRT-AKT axis regulation might be complex and tissue-dependent. However, the correlation between CRT and PI3K/Akt pathway in HCC has not been well addressed. Our data showed that CRT knockdown obviously decreased the phosphorylation level of Akt in HCC cells, indicating CRT might regulate the above cellular processes of HCC via PI3K/Akt signaling pathway. The findings are consistent with the results obtained in some other type of tumors and may provide a potential mechanism for the role of CRT in HCC. It is important to note that much work is needed to understand the details of the CRT mediated activation of the Akt pathway. Potential mechanisms may involve CRT’s chaperoning effects and endoplasmic reticulum stress response, or direct interactions between CRT and factors in the Akt pathway through certain unknown CRT functions.

## Conclusions

CRT affects the biological phenotypes of hepatocellular carcinoma, likely via the PI3K/Akt pathway. CRT may be a potential biomarker and therapeutic target of the hepatocellular carcinoma.
